# Parent-Offspring Transmission of Adipocytokine Levels and Their Associations with Metabolic Traits

**DOI:** 10.1371/journal.pone.0018182

**Published:** 2011-04-04

**Authors:** Nasser M. Al-Daghri, Omar S. Al-Attas, Majed S. Alokail, Khalid M. Alkharfy, Sobhy M. Yakout, Shaun B. Sabico, Greg C. Gibson, George P. Chrousos, Sudhesh Kumar

**Affiliations:** 1 Biomarkers Research Program, Biochemistry Department, College of Science, King Saud University, Riyadh, Kingdom of Saudi Arabia; 2 Clinical Pharmacy Department, College of Pharmacy, King Saud University, Riyadh, Kingdom of Saudi Arabia; 3 Center for Integrative Genomics, School of Biology, Georgia Institute of Technology, Atlanta, Georgia, United States of America; 4 Division of Endocrinology, Metabolism and Diabetes, University of Athens Medical School, Children's Hospital Aghia Sophia, Athens, Greece; 5 Diabetes and Metabolism Unit, Clinical Sciences Research Institute, Warwick Medical School, University of Warwick, Coventry, United Kingdom; Florida International University, United States of America

## Abstract

Adipose tissue secreted cytokines (adipocytokines) have significant effects on the physiology and pathology of human metabolism relevant to diabetes and cardiovascular disease. We determined the relationship of the pattern of these circulating hormones with obesity-related phenotypes and whether such pattern is transmitted from parent to offspring. A combined total of 403 individuals from 156 consenting Saudi families divided into initial (119 families with 123 adults and 131 children) and replication (37 families with 58 adults and 91 children) cohorts were randomly selected from the RIYADH Cohort study. Anthropometrics were evaluated and metabolic measures such as fasting serum glucose, lipid profiles, insulin, leptin, adiponectin, resistin, tumor necrosis factor alpha (TNFα), activated plasminogen activator inhibitor 1 (aPAI1), high sensitivity C-reactive protein (hsCRP) and angiotensin II were also assessed. Parent-offspring regressions revealed that with the exception of hsCRP, all hormones measured showed evidence for significant inheritance. Principal component (PC) analysis of standardized hormone levels demonstrated surprising heritability of the three most common axes of variation. PC1, which explained 21% of the variation, was most strongly loaded on levels of leptin, TNFα, insulin, and aPAI1, and inversely with adiponectin. It was significantly associated with body mass index (BMI) and phenotypically stronger in children, and showed a heritability of ∼50%, after adjustment for age, gender and generational effects. We conclude that adipocytokines are highly heritable and their pattern of co-variation significantly influences BMI as early as the pre-teen years. Investigation at the genomic scale is required to determine the variants affecting the regulation of the hormones studied.

## Introduction

The rising epidemic of overweight and obesity among developing nations has been well established in several epidemiological studies of different ethnic groups from all over the world [1]–[3]. However, reductions in the over-all prevalence secondary to non-significant trends in obesity patterns have been demonstrated in several industrialized nations [4]–[7]. In the Kingdom of Saudi Arabia (KSA), the most recent study conducted to determine the baseline national prevalence of childhood obesity indicated an over-all prevalence of 9.3%, considered intermediate between developing and industrialized nation [8]. It is important to stress that the indigenous Saudi population seems to have an increased genetic predisposition to develop diabetes mellitus type 2 (DM T2), which is further amplified by increased prevalence of obesity, high rates of consanguinity, and the presence of other components of the metabolic syndrome (MetS) [9]. The features of MetS include insulin resistance, glucose intolerance, hypertension, dyslipidemia, and central obesity, all of which are risk factors for coronary heart disease (CHD) and DM T2. The rapid industrializations in KSA and other developing nations have been accompanied by increased prevalence of MetS even among younger populations [10].

Aside from the traditional measurements used to diagnose obesity and MetS, several key novel biomarkers, known as adipocytokines or adipokines, have been associated with the regulation of body fat and over-all human metabolism. Increased leptin, the satiety hormone, and decreased adiponectin, the insulin sensitizing adipocytokine, have been well established among those with increased body fat [11]–[12]. Resistin, the adipocytokine initially linked to obesity and insulin resistance in humans [13], has been shown to be more related to inflammation and has been associated with cardiovascular risk factors, including hypertension [14]–[16]. Increased levels of activated plasminogen activator inhibitor 1 (aPAI-1), tumor necrosis factor-alpha (TNFα), C-reactive protein (CRP), as well as other conditions such as obesity and the MetS, are now considered low-grade inflammatory states [17]. Lastly, angiotensin II, which is the effector peptide of the renin-angiotensin system (RAS), has regained the spotlight for its role in several components of the metabolic syndrome, including insulin resistance, hypertension and body weight regulation [18], [19].

We hypothesize that individual adults harboring metabolic alterations, as manifested by change of the biomarkers mentioned above, transmit the same patterns of biomarkers to their offspring. Several recent studies appear to support this hypothesis [20]–[22]. If this is the case, then there is an urgent need to look for novel biomarkers that may improve prediction of future risk for chronic non-communicable diseases and allow understanding of the underlying mechanisms that result in co-regulation of various biomarkers. To the best of our knowledge, no study has as yet analyzed a multitude of several metabolic phenotypes in terms of heritability and association to key components of the MetS. The purpose of this study was therefore to determine heritability patterns among biomarkers of obesity, insulin resistance and other markers of the MetS by measuring parent-offspring regressions in subjects of Saudi Arab origin. This measure of heritability does not necessarily imply a genetic mechanism, but does indicate considerable familial structure to adipocytokine expression related to MetS.

## Materials and Methods

### Ethical Statement

Written and informed consents were obtained from all participants including parents, and assent from the children prior to inclusion. Ethical approval was obtained from the research ethics committee of the College of Medicine Research Center (CMRC) in King Khalid University Hospital, Riyadh, KSA.

### Subjects

This cross-sectional study was carried out at the Biomarkers Research Program, King Saud University (KSU), Riyadh, KSA. A total of 254 subjects or 119 families (123 adults and 131 children) were randomly selected from the RIYADH COHORT, a capital-wide study supported by the Ministry of Health and King Saud University for the screening of novel biomarkers of more than 16,000 Saudi subjects (aged 1–80 years) recruited from different primary care centers all over Riyadh, KSA. Families were randomly selected from the database using the Microsoft Excel function. Subjects, who were on medications for diabetes and hypertension without complications (*e.g.* diabetic complications, coronary artery disease, liver or kidney failure), were included to avoid selection bias. Patients were asked to complete general questionnaires, which included medical history.

### Anthropometrics

Anthropometrics was carried out by a designated nurse and physician, as part of an on-going research program ascertaining: height (to the nearest 0.5 cm), weight (to the nearest 0.1 kg), waist and hip circumferences (measured using a standardized measuring tape in cm), in addition to systolic and diastolic blood pressure measurements. BMI was calculated as kg/m^2^. Obesity for adults was defined as having a BMI of ≥30 kg/m^2^, while overweight was defined as a BMI of >25 but <30 kg/m^2^. Classification of obesity for children was based in the international age and gender-specific criteria proposed by Cole and colleagues [23].

### Biochemical Measurements

Morning fasting blood samples were taken previously from the on-going RIYADH COHORT and were kept in the biobank of the Biomarkers Research Program (BRP) at KSU. Serum glucose and lipid profile were determined using routine laboratory methods. Serum insulin, leptin, adiponectin, resistin, TNFα and aPAI-1 were quantified using multiplex assay kits that utilize fluorescent microbead technology, allowing simultaneous quantification of several target proteins within a single serum sample of 50–100 µL [24]. These included pre-mixed and fully customized panels that utilize the Luminex® xMAP® Technology platform (Luminexcorp, TX, USA). For parameters measured using the multiplex assay, the intra-assay variation was 1.4-7.9% and inter-assay variation of <21%. Minimum detectable concentrations (MDC) were as follows: insulin, 50.9 pg/ml; leptin, 85.4 pg/ml; adiponectin, 145.4 pg/ml; resistin, 6.7 pg/ml; TNFα, 0.14 pg/ml, and PAI-1, 1.3 pg/ml. hsCRP was determined using enzyme-linked immunosorbent assays (ELISA) (Immunodiagnoztik AG, Bensheim, Germany) with an intra-assay variability of 5.5–6.0% and inter-assay variation of 11.6–13.8%. Angiotensin II (ANG II) was quantified using fluorescent-based non-radioactive immunoassay (MDC 13pg/ml; linear range 13–240 pg/ml) (Phoenix Pharmaceuticals, CA, USA). All fasting samples fell within the detection range.

### Replication Cohort

An additional cohort of 149 Saudi individuals or 37 families (58 adults, 91 children) was randomly selected from the master database of RIYADH COHORT for generation of a replication dataset.

### Statistical analyses

Descriptive statistics reported in Table 1 were generated using SPSS version 11.5 (Chicago, IL). Frequencies were presented as percentage, while continuous variables that assumed normality was shown as mean ± standard deviation. Medians (inter-quartile range) were shown for non-normal continuous variables. Independent Student t-test was used to compare gender differences for normal parameters and chi-square for frequencies. For non-normally distributed parameters, the Mann-Whitney U-test was utilized for comparisons. All subsequent analyses were performed using SAS/JMP version 4 (Cary, North Carolina). Pearson correlation and regression analyses were performed on standardized residuals from linear models of systolic and diastolic blood pressure, the anthropomorphic measures, or log-transformed measures of glucose, triglycerides, cholesterols, insulin, leptin, adiponectin, resistin, TNFα, ANG II, aPAI1 and hsCRP that included covariates representing cohort, gender, generation, and age within generation. Principal component analysis was performed on the correlations among the standardized residuals for the seven adipocytokines.

**Table 1:** General Characteristics of Parents and Children.

Parameter	Parents	Children
N	123	131
Obesity (%)	47.9	29.7
DM T2 (%)	30.6	0
Hypertension (%)	14.0	0
Age (years)	45.9±10.1	9.37±2.6
*Anthropometrics*		
BMI (kg/m^2^)	30.0±5.6	20.0±5.5
Waist circumference (cm)	95.4±13.3	62.8±5.7
Hips circumference (cm)	104.9±6	72.2±15.0
Systolic BP (mmHg)	121.2±12.6	99.8±15.0
Diastolic BP (mmHg)	79.6±9.1	66.0±8.0
*Metabolites*		
Glucose (mmol/L)	7.1±3.7	4.9±1.0
Triglycerides (mmol/L)#	1.5 (1.03–2.5)	0.88 (0.7–1.2)
Total Cholesterol (mmol/L)	5.2±1.1	4.4±0.9
LDL-Cholesterol (mmol/L)	3.4±0.9	2.8±0.7
HDL-Cholesterol (mmol/L)	0.86±0.23	1.1±0.29
*Hormones*		
Insulin (IU/ml)#	8.3 (5.6–13.0)	5.9 (3.6–9.1)
Leptin (ng/ml)#	11.9 (5.3–18.6)	4.5 (1.7–13.7)
Adiponectin (µg/ml)#	8.4 (6.2–14.7)	19.6 (13.6–28.0)
Resistin (ng/ml)#	18.6 (13.8–25.3)	16.2 (12.3–23.2)
TNFα (pg/ml)#	2.9 (2.1–4.3)	3.9 (2.6–5.3)
aPaI-1 (pg/ml)#	7.3 (1.7–18.8)	11.6 (4.4–21.4)
ANG II (ng/ml)#	0.54 (0.43–0.75)	0.57 (0.44–0.73)
hsCRP (µg/ml)#	3.2 (1.1–7.0)	0.9 (0.28–3.4)

**Note**: Data is presented as % for frequency; mean ± standard deviation for normally distributed data; # denotes non-Gaussian distribution and thus presented as median (inter-quartile range).

## Results

The general characteristics of both parents and children are presented in Table 1. There was a high prevalence of obesity in both cohorts, and a high prevalence of T2DM and hypertension among parents. The concentrations of eight hormones (insulin, leptin, adiponectin, resistin, TNFα, ANG II, aPAI1 and hsCRP), as well as the various other metabolic measurements, were determined in the initial cohort of 254 individuals, consisting of 119 parent-offspring pairs as well as 12 additional siblings and 2 parents. After adjustment for gender and generational effects, BMI was significantly influenced by five hormones (leptin, insulin, adiponectin, aPAI1 and hsCRP). Furthermore, the first principal component of the hormones also influenced BMI and showed evidence for significant heritability. Consequently, we sought to replicate these results with our second cohort consisting of 149 individuals from 37 families (21 with both parents), with an average of 2.5 children per family. Because the sub-cohort analysis provided similar results, a combined analysis of all 403 individuals from 156 families was undertaken; any discrepancies between cohorts are addressed within the text. Supplementary tables provide the analyses for each cohort separately.

### Heritability of Metabolic Factors

**Figure 1:**
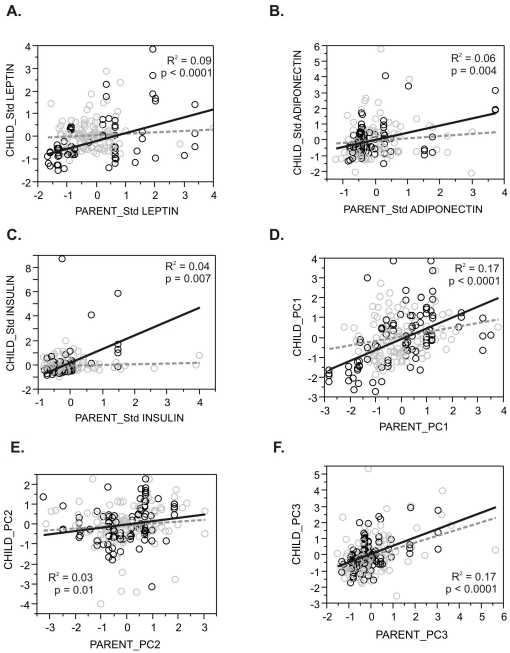
Parent-Offspring Regressions. Each plot shows the regression for the indicated adipocytokine measure of the child against the parent or mid-parent (where both parents were measured). Each measure is normalized and adjusted for generation, gender, cohort, and age within generation. Gray circles and dashed line indicate the initial cohort (generally single parent-child pairs) and the black circles and solid line indicate the replication cohort (generally larger families). (A) Leptin. (B) Adiponectin. (C) Insulin. (D) Principal Component 1. (E) PC2. Note that removal of a dozen outliers with high levels of resistin results in a much stronger regression. (F) PC3. Regression coefficients and significance are indicated in Supplementary Tables, and in Table 2 for the combined dataset. R-squared and p-values correspond to the combined dataset as documented in Table 2.

Parent-offspring regressions, which provide an estimate of heritability, were estimated for serum concentrations of eight hormones, five metabolites, and anthropometric parameters, as shown in Table 2 (initial and replicate cohort parent-offspring regressions are shown in Table S1 and S2, respectively). Each of the variables measured, with the exception of hsCRP, showed evidence for highly significant inheritance, as did all three cholesterol measures (total, HDL-, and LDL-cholesterol). The leptin, insulin, and adiponectin parent-offspring regressions were only significant in the replication cohort (Figure 1), suggesting that measuring both parents as well as multiple children increased the power of this analysis; only aPAI1 showed reduced significance in the replication cohort. BMI was also significant in both cohorts, although the regression explained less than 10 percent of the phenotypic variance. Heritability was estimated as twice the slope of the regression in the initial cohort, where only one parent was available, and as the slope of the regression in the replication cohort: with considerable error it ranges between ∼25% (leptin) and ∼63% (TNFα).

**Table 2:** Unadjusted Parent-Offspring Regressions for Raw Trait Measures.

Trait	Slope	R^2^	N pairs	*P*-Value
BMI (kg/m^2^)	0.30	0.06	207	0.0002
Glucose (mmol/L)	0.06	0.00	196	0.21
Triglycerides (mmol/L)	0.06	0.02	196	0.04
Total Cholesterol (mmol/L)	0.32	0.17	195	2×10^−9^
LDL-Cholesterol (mmol/L)	0.34	0.21	194	1×10^−11^
HDL-Cholesterol (mmol/L)	0.43	0.15	196	2×10^−8^
Leptin (ng/ml)	0.25	0.09	175	5×10^−5^
Insulin (IU/ml)	0.29	0.04	176	0.007
Adiponectin (µg/ml)	0.31	0.06	200	0.004
Resistin (ng/ml)	0.40	0.21	196	6×10^−12^
TNFα (pg/ml)	0.58	0.26	169	7×10^−13^
aPaI-1 (pg/ml)	0.41	0.17	180	4×10^−9^
ANG II (ng/ml)	0.45	0.10	172	1×10^−5^
hsCRP (µg/ml)	0.05	0.00	195	0.45
Principal Component 1	0.43	0.17	200	1×10^−9^
Principal Component 2	0.16	0.03	200	0.010
Principal Component 3	0.45	0.17	200	6×10^−10^
Adj. Height (cm)	0.24	0.03	199	0.006
Adj. Weight (kg)	0.22	0.04	200	0.003
Adj. Hip circumference (cm)	0.46	0.21	192	1×10^−11^
Adj. Waist circumference (cm)	0.44	0.21	195	2×10^−11^
Waist-to-hip ratio	0.12	0.01	194	0.18
Systolic blood pressure (mmHg)	0.10	0.01	173	0.16
Diastolic blood pressure (mmHg)	−0.06	0.00	173	0.44

**Note:** Adj  =  adjusted; Significant at *P*<0.05.

In order to assess whether adipocytokines are co-regulated in a heritable manner, principal component (PC) analysis was performed on the standardized hormone levels. Surprising heritability of the three most common axes of variation, which jointly explained 51% of the variation, was observed. PC1 (21%) was most strongly loaded on levels of leptin, TNFα, insulin, and aPAI1, and inversely with adiponectin, and showed a heritability of ∼50%, which is at least as large as that of the individual contributing hormones. Table 3 indicates that these loadings are consistent in both cohorts and in the combined analysis, with the exception of Angiotensin II, which does not co-vary consistently with the metabolism-regulating hormones. PC2 (15%) is less significantly transmitted from parent to offspring, largely because 20 individuals had elevated levels of resistin, TNFα, or hsCRP that influence the loadings for this component (Figure 1B). PC3 (15%) was as heritable as PC1, and loaded adiponectin, aPAI1 and TNFα against leptin, insulin, and CRP.

**Table 3:** Principal Component (PC) Loadings.

	PC1			PC2			PC3		
	Init	Rep	Total	Init	Rep	Total	Init	Rep	Total
Adiponectin	−0.27	−0.46	−0.29	0.11	0.26	0.31	0.56	0.41	0.59
ANG II	−0.42	0.45	0.20	0.46	−0.17	−0.28	0.01	0.26	0.20
aPAI1	0.56	0.20	0.39	−0.05	0.44	0.61	0.34	0.51	0.06
hsCRP	0.04	0.11	0.28	0.46	0.39	−0.54	−0.43	−0.64	0.22
Insulin	0.31	0.21	0.33	0.21	0.31	−0.18	−0.32	−0.15	−0.09
Leptin	0.43	0.46	0.51	0.14	0.25	−0.02	−0.21	−0.25	−0.20
Resistin	−0.02	−0.24	0.16	0.63	0.62	−0.15	0.18	−0.01	0.70
TNF-α	0.40	0.47	0.50	0.30	0.09	0.31	0.45	0.36	0.13

**Note:** Data are presented as eigenvalues; Init  =  Initial Cohort; Rep  =  Replication Cohort.

### Correlation of Metabolic Factors with Adipocytokines

We next asked whether any of the hormones measured correlated with metabolic phenotypes by fitting linear models with cohort, generation, gender, and age within generation as covariates. The significance of the effect of each hormone or principal component, as well as the percent variance explained by each model, is shown in Table 4. These values should be contrasted with the null model (top line) that only includes effects of age, gender, and generation. Angiotensin II was a highly significant predictor of HDL-cholesterol, and both adiponectin and insulin correlate modestly with total triglycerides, but no other tests were significant after adjustment for multiple comparisons. BMI associated highly significantly with leptin, aPAI1, and CRP (and to a lesser extent adiponectin and insulin), as well as with PC1, which (like leptin) explains as much as 10% of the variance of BMI on top of the age, gender and generational effects. These results were quite consistent between the two cohorts.

**Table 4:** Adipocytokine Correlations with Metabolic Measures.

	BMI		Total Cholesterol		HDL-C		LDL-C		Triglycerides	
	R^2^	Sig	R^2^	Sig	R^2^	Sig	R^2^	Sig	R^2^	Sig
Null	0.51		0.15		0.10		0.15		0.17	
PC1	0.61	5×10^−21^	0.15	NS	0.11	NS	0.15	NS	0.17	NS
PC2	0.51	0.05	0.15	NS	0.11	NS	0.16	NS	0.17	NS
PC3	0.51	NS	0.15	NS	0.12	0.07	0.15	NS	0.18	0.02
Leptin	0.65	1×10^−23^	0.18	NS	0.11	NS	0.17	NS	0.17	NS
Insulin	0.54	0.0031	0.17	NS	0.13	0.04	0.17	NS	0.19	0.002
TNFα	0.53	NS	0.18	NS	0.11	NS	0.17	NS	0.16	NS
Adiponectin	0.52	0.0003	0.16	NS	0.12	0.06	0.15	NS	0.18	0.006
aPAI1	0.54	2×10^−5^	0.15	NS	0.09	NS	0.15	NS	0.16	NS
Resistin	0.51	NS	0.15	NS	0.11	NS	0.15	NS	0.17	NS
hsCRP	0.54	6×10^−8^	0.15	NS	0.13	0.02	0.16	NS	0.17	NS
ANG II	0.52	NS	0.15	NS	0.15	5×10^−5^	0.15	NS	0.19	NS

**Note:** Table shows percent variance explained (R^2^) by the full model including Cohort, Generation, Gender, and Age(Gender), as well as the indicated adipocytokine (or principal component) with BMI or metabolite levels, as well as the significance of the adipocytokine/PC term. The first row labeled Null is the model without an adipocytokine/PC, and shows the variance explained jointly by Cohort, Generation, Gender and Age (Gender) for comparison.

**Figure 2:**
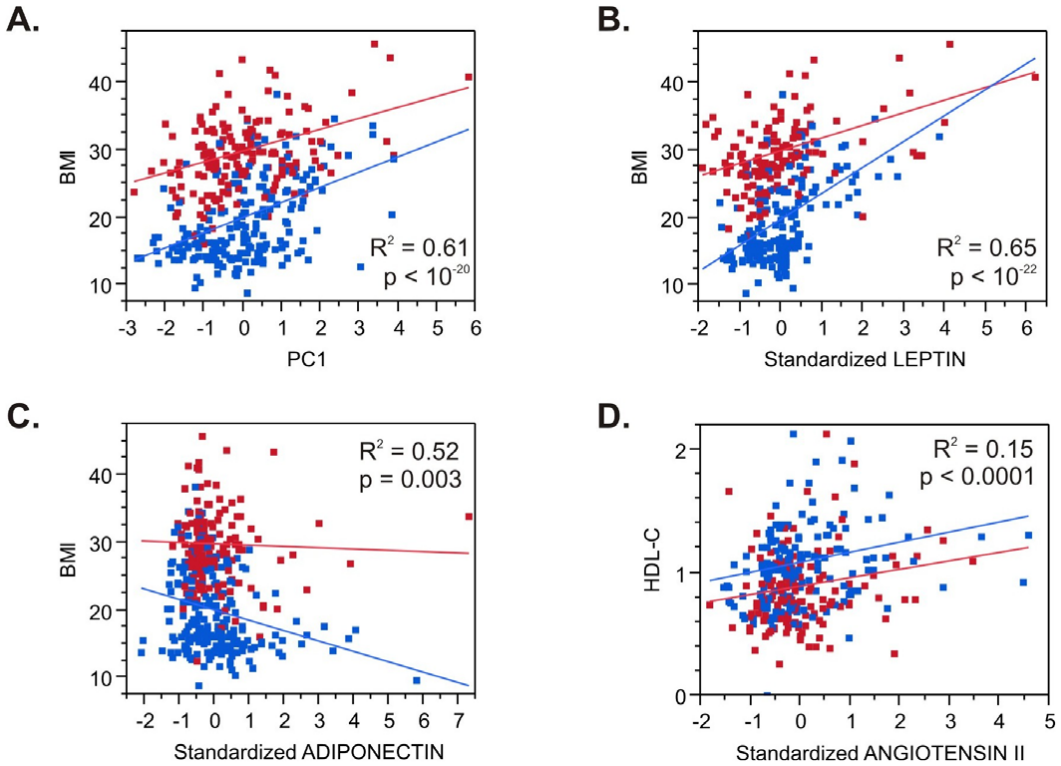
Adipocytokine – Phenotype Correlations. Each plot shows the regression of the indicated phenotype (A–C: Body Mass Index; D: serum HDL-Cholesterol) against the standardized measure of adipocytokine (B: Leptin; C: Adiponectin; D: Angiotensin II) or PC1 (A). Red points are the parents, blue points the children. Note that in each case the correlation is stronger in the children, and that although the regressions for PC1 and leptin explain similar proportions of the variance, as indicated in Table 3, the PC1 values cover a more even spread of values and are more consistent across the generations. R-squared and p-values correspond to the full model as documented in Table 4.

Most of these correlations were considerably stronger in the children than adults, as noted in Figure 2. PC1 explains 22% of BMI in children (p<3×10^−15^), and 15% in adults (p<2×10^−7^), and similarly, leptin (31%, p<5×10^−20^ in children; 17%, p<5×10^−8^ adults) was strongly associated with BMI. The aPAI1, insulin, and adiponectin effects were only significant in the children, as was the angiotensin influence on HDL cholesterol (8% explained, p<0.0005), though all these effects trended in the same direction in adults.

### Familial Segregation of Abnormal Adipocytokine Profiles

**Figure 3:**
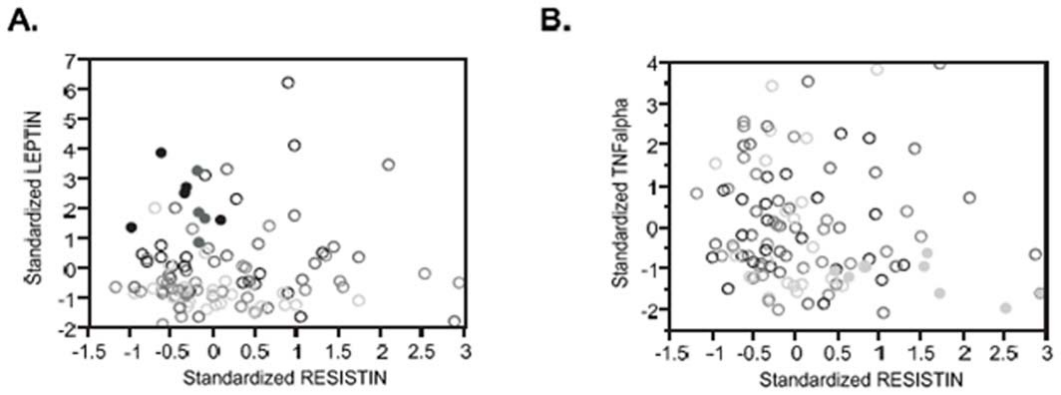
Clustering of Phenotypes in Families. Several families in the replication cohort were observed where each of the parents and their children were in the upper or lower quartile for age-adjusted BMI, as well as for two or more adipocytokine measures. Shading of each circle corresponds to age-adjusted BMI (low BMI, light; high BMI dark), and the filled circles represent members of two families in each plot. (A) All members of families 124 and 128 were obese and had high leptin coupled with low resistin concentrations. (B) All members of families 157 and 160 had low TNFα but high resistin concentrations and were in the lowest third of age-adjusted BMI.

Given the strong evidence for parent-offspring correlation, as well as association of adipocytokines with BMI, we next asked whether specific families in the replication cohort showed aberrant profiles for specific hormones, where each member of the family lies in the upper or lower quartile for two or more measures. Two examples involving resistin are illustrated in Figure 3. Families 157 and 160 had relatively high resistin but low TNFα levels (and 157 also had low aPAI1); Families 129 and 148 had low resistin but high insulin; and families 124 and 128 had low resistin coupled with high leptin levels. Correspondingly, 4 of 5 members of family 128 were in the upper quintile for BMI and lower quintile for HDL-C (the fifth child was just 10 years old at time of sampling), and for family 124 HDL was uniformly low while the parents and older child were obese. By contrast, members of families 157 and 160 all had BMI measures in the lower half for their generation.

## Discussion

Recent epidemiologic evidence using the same ethnic group in this study highlight a dysmetabolic pattern that was observed to be common in both adults [25] and children [10], suggesting that not only childhood weight gain but also abnormal biochemical profiles associated with the components of the metabolic syndrome, are highly heritable [26], [27]. While results from the present study involving a sample of 403 Saudi subjects strengthen these hypotheses, the major novel finding is that various adipocytokines and inflammatory markers also show highly significant parent-offspring regressions which, consequently, also correlate significantly with BMI. Several studies involving leptin [26], [28], adiponectin [29], [30] and resistin [31], have individually shown pleiotropic effects to obesity-related phenotypes with significant heritability estimates. To the best of our knowledge however, this is the first study to demonstrate inherited metabolic patterns involving a multitude of adipocytokines and inflammatory markers in an at risk population.

In the present study, the hormone-phenotype correlations are stronger in children between the ages of 5 and 18 as opposed to their parents. Since BMI and other obesity-related phenotypes are largely dictated by a stronger genetic influence from early years to young adulthood [32], [33], our results suggest that the expression of obesity-related hormones used in this study follow the same genetic influence in this period, with environmental factors probably playing an increasingly important role later in life. In fact, the somewhat weaker correlations with BMI among parent subjects (adults) are consistent with the weakening of any mechanism of genetic regulation under the influence of cumulative environmental exposures, as individuals' age [20], [34]. Furthermore, aside from the dietary and behavioral factors inducing a pro-obesity adipocytokine profile in adults, it is possible that this condition is established early in life in the at risk population, and modified as people age. While large-scale studies of obesity involving parent-offspring subjects overwhelmingly indicate an increased risk to childhood obesity among those having overweight parents [35]–[37], None of these studies actually showed whether genetic predisposition to obesity by itself will translate to an equally heritable obesity-related abnormal metabolic profile that manifests as early as pre-teens. Our study is the first to reveal that the pattern of covariance in adipocytokine abundance, as clustered in the first major principal component, was not only highly predictive of body mass index, but also significantly transmitted from parent to offspring.

The strong correlation of the hormones studied with BMI and HDL-cholesterol in children between the ages of 5 and 18 is noteworthy from the perspective of the global epidemic of childhood obesity. This was observed not only for individual hormones, but also for the first principal component, suggesting that whatever mechanism accounts for the co-regulation is operating early in life. Particularly noteworthy is the significantly stronger association of ANG II to HDL-cholesterol in children, mechanisms which might involve translocation of scavenger receptor type-BI (SR-BI) in the adipose tissue [38], or angiotensin-converting enzyme (ACE) polymorphisms that were recently linked to components of the metabolic syndrome including HDL-cholesterol [39], both of which require further investigation.

Particularly in human studies, caution is required in interpreting heritability estimates from parent-offspring regression as strong evidence for a genetic component, since family members share cultural and social environments. Genotype-based studies would be required to establish that genetic variation influences adipocytokine levels. Genome-wide association studies (GWAS) have uncovered several loci that affect BMI [40], [41]. Our results establish the minimal requirements for GWAS of adipocytokine levels as potential mediators of increased body mass and subsequent susceptibility to chronic non-communicable diseases including DM T2. This strategy would be particularly revealing if it uncovered variants affecting the covariance of leptin, TNFα, insulin, aPAI1 and adiponectin, in particular, that generates PC1. Indeed, while adipocytokine effects have been well studied in adults, these results call for more extensive studies of their impact on metabolic physiology that encompasses even pre-teenage children.

In conclusion, this study highlights the existence not just of individuals, but of families that show somewhat unusual combinations of adipocytokines that are likely to contribute to elevated risk of chronic diseases. These families might have had specific environmental exposures that lead to the aberrant patterns, but the possibility that segregation of genetic polymorphisms is responsible should be pursued. Further studies at the genomic scale are required, examining individuals with extreme phenotypes, as the power of whole genome sequencing promises to identify not only mutations of large effect, but also target genes that can be utilized for the development of customized pharmacologic interventions.

## Supporting Information

Table S1 Parent-Offspring Regressions for Raw Trait Measures in the Initial Cohort.
(DOC)

Table S2 Parent-Offspring Regressions for Raw Trait Measures in the Replication Cohort.
(DOC)
