# Supplementary material for: Parent-Offspring Transmission of Adipocytokine Levels and Their Associations with Metabolic Traits
Source: PLoS One. 2011 Apr 4;6(4):e18182. doi: 10.1371/journal.pone.0018182 (PMC3070726; doi:10.1371/journal.pone.0018182)
Supplement: Table S1 — Parent-Offspring Regressions for Raw Trait Measures in the Initial Cohort. (DOC) [file pone.0018182.s001.doc]

**Table S1.** Parent-Offspring Regressions for Raw Trait Measures in the Initial Cohort

| **Trait** | **Slope** | **R^2^** | **N pairs** | ***P*-Value** |
| --- | --- | --- | --- | --- |
| BMI (kg/m^2^) | 0.25 | 0.05 | 117 | 0.01 |
| Glucose (mmol/L) | 0.08 | 0.00 | 116 | 0.29 |
| Triglycerides (mmol/L) | 0.06 | 0.03 | 116 | 0.04 |
| Total Cholesterol (mmol/L) | 0.21 | 0.06 | 115 | 0.007 |
| LDL-Cholesterol (mmol/L) | 0.24 | 0.11 | 114 | 2x10^-4^ |
| HDL-Cholesterol (mmol/L) | 0.13 | 0.01 | 116 | 0.27 |
| Leptin (ng/ml) | 0.07 | 0.00 | 108 | 0.42 |
| Insulin (IU/ml) | 0.06 | 0.00 | 108 | 0.30 |
| Adiponectin (µg/ml) | 0.15 | 0.01 | 109 | 0.29 |
| Resistin (ng/ml) | 0.39 | 0.24 | 109 | 5x10^-8^ |
| TNF-α (pg/ml) | 0.22 | 0.04 | 101 | 0.02 |
| aPaI-1 (pg/ml) | 0.46 | 0.27 | 89 | 8x10^-8^ |
| Angiotensin II (ng/ml) | 0.56 | 0.09 | 84 | 0.003 |
| C-Reactive Protein (µg/ml) | 0.09 | 0.00 | 106 | 0.36 |
| Principal Component 1 | 0.23 | 0.04 | 109 | 0.02 |
| Principal Component 2 | 0.16 | 0.02 | 109 | 0.06 |
| Principal Component 3 | 0.43 | 0.15 | 109 | 2x10^-5^ |
| Adj. Height | 0.24 | 0.04 | 109 | 0.02 |
| Adj. Weight | 0.19 | 0.04 | 109 | 0.03 |
| Adj. Hip circumference | 0.42 | 0.19 | 106 | 2x10^-6^ |
| Adj. Waist circumference | 0.26 | 0.06 | 107 | 0.006 |
| Waist-to-hip ratio | 0.22 | 0.10 | 115 | 0.001 |
| Systolic blood pressure | 0.12 | 0.01 | 96 | 0.23 |
| Diastolic blood pressure | 0.00 | 0.00 | 96 | 0.87 |

**Note:** *P*-value significant at < 0.05
