# Supplementary material for: Parent-Offspring Transmission of Adipocytokine Levels and Their Associations with Metabolic Traits
Source: PLoS One. 2011 Apr 4;6(4):e18182. doi: 10.1371/journal.pone.0018182 (PMC3070726; doi:10.1371/journal.pone.0018182)
Supplement: Table S2 — Parent-Offspring Regressions for Raw Trait Measures in the Replication Cohort. (DOC) [file pone.0018182.s002.doc]

**Table S2.** Parent-Offspring Regressions for Raw Trait Measures in the Replication Cohort

| **Trait** | **Slope** | **R-Squared** | **N pairs** | ***P*-Value** |
| --- | --- | --- | --- | --- |
| BMI (kg/m^2^) | 0.42 | 0.09 | 90 | 0.003 |
| Glucose (mmol/L) | 0.03 | 0.00 | 80 | 0.53 |
| Triglycerides (mmol/L) | 0.09 | 0.00 | 80 | 0.33 |
| Total Cholesterol (mmol/L) | 0.39 | 0.26 | 80 | 7x10^-7^ |
| LDL-Cholesterol (mmol/L) | 0.43 | 0.28 | 80 | 3x10^-7^ |
| HDL-Cholesterol (mmol/L) | 0.62 | 0.40 | 80 | 3x10^-10^ |
| Leptin (ng/ml) | 0.35 | 0.20 | 68 | 1x10^-4^ |
| Insulin (IU/ml) | 1.13 | 0.15 | 68 | 7x10^-4^ |
| Adiponectin (µg/ml) | 0.46 | 0.18 | 91 | 2x10^-5^ |
| Resistin (ng/ml) | 0.50 | 0.17 | 87 | 5x10^-5^ |
| TNF-α (pg/ml) | 0.82 | 0.43 | 68 | 6x10^-10^ |
| aPaI-1 (pg/ml) | 0.46 | 0.27 | 91 | 8x10^-8^ |
| Angiotensin II (ng/ml) | 0.41 | 0.12 | 88 | 6x10^-4^ |
| C-Reactive Protein (µg/ml) | 0.02 | 0.00 | 89 | 0.83 |
| Principal Component 1 | 0.55 | 0.28 | 91 | 5x10^-8^ |
| Principal Component 2 | 0.16 | 0.03 | 91 | 0.08 |
| Principal Component 3 | 0.52 | 0.22 | 91 | 2x10^-6^ |
| Adj. Height | 0.10 | 0.01 | 90 | 0.17 |
| Adj. Weight | 0.29 | 0.05 | 91 | 0.03 |
| Adj. Hip circumference | 0.50 | 0.21 | 86 | 5x10^-6^ |
| Adj. Waist circumference | 0.55 | 0.34 | 88 | 2x10^-9^ |
| Waist-to-hip ratio | -0.11 | 0.00 | 81 | 0.47 |
| Systolic blood pressure | 0.08 | 0.00 | 77 | 0.44 |
| Diastolic blood pressure | -0.05 | 0.00 | 77 | 0.78 |
